# Anterolateral thigh free flap using modified turbocharging method: a case report

**DOI:** 10.3389/fsurg.2024.1273843

**Published:** 2024-10-01

**Authors:** Yooseok Ha, Donghyun Kim, Hyeokjae Kwon, Sunje Kim, Seung Han Song, Sang-Ha Oh, Joo-hak Kim, Ho Jik Yang, Hyunwoo Kyung

**Affiliations:** 1 Department of Plastic and Reconstructive Surgery, Chungnam National University Hospital, Daejeon, Republic of Korea; 2 Department of Plastic and Reconstructive Surgery, College of Medicine, Chungnam National University, Daejoen, Republic of Korea; 3 Department of Plastic and Reconstructive Surgery, Chungnam National University Sejong Hospital, Sejong, Republic of Korea

**Keywords:** turbocharging, anterolateral thigh, soft tissue defect reconstruction, perforator flap, salvage technique

## Abstract

The free flap is a versatile option for reconstruction of soft tissue defects around the ankle. In patients with poor lower leg circulation, arterial insufficiency is one of the complications that can occur immediately after vessel anastomosis during free flap surgery. The authors were able to improve blood circulation in the flap by using modified turbocharging method in which another perforator was anastomosed to the distal end of the main pedicle.

## Purpose

Soft tissue defects around the ankle are a challenge for reconstructive surgery. If the defect size is small in the lower one-third of the lower limb or around the ankle, local flaps such as a reverse sural flap or a propeller flap can be tried. However, if the defect is large, it is often necessary to perform a free flap (1). The arterial insufficiency is often encountered after anastomosis of the perforator and pedicle, especially in the case of poor blood circulation in the lower legs (2). The authors performed an anterolateral thigh (ALT) free flap to reconstruct a defect around the ankle in a patient with poor blood circulation in the lower limb. After vessel anastomosis, arterial blood flow was not achieved with the flap, and arterial insufficiency could be improved by performing anastomosis with the distal end of the pedicle on the remaining perforator.

## Case review

**Figure 1:**
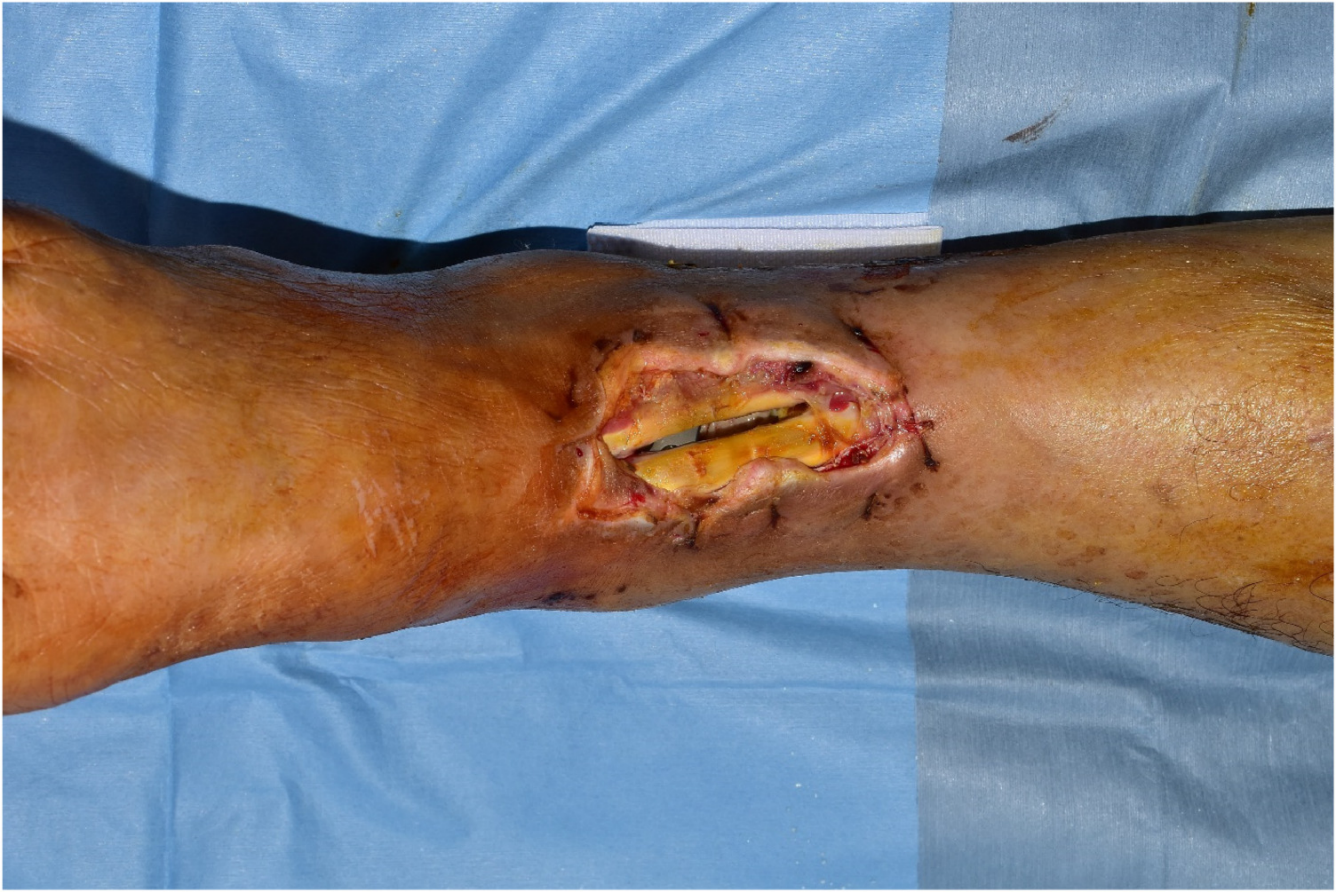
Preoperative photo. Tendons and metal plates are exposed.

A 72-year-old male patient visited the hospital with the right tibiofibular fracture. One day after visiting the hospital, the orthopedic department applied closed reduction and external fixation, and one week later, open reduction and internal fixation was performed. Skin necrosis occurred in the ankle area, and after the implementation of the debridement, soft tissue defect occurred and was referred to a plastic surgery clinic (Figure 1).

ALT free flap was planned and computed tomography angiography was performed. In the reading, there was diffuse atherosclerosis, both distal superficial femoral arteries, and the blood supply below the ankle was not smooth. After peripheral angiogram and balloon angioplasty, occlusion of the anterior tibial artery (ATA) was recanalized.

**Figure 2:**
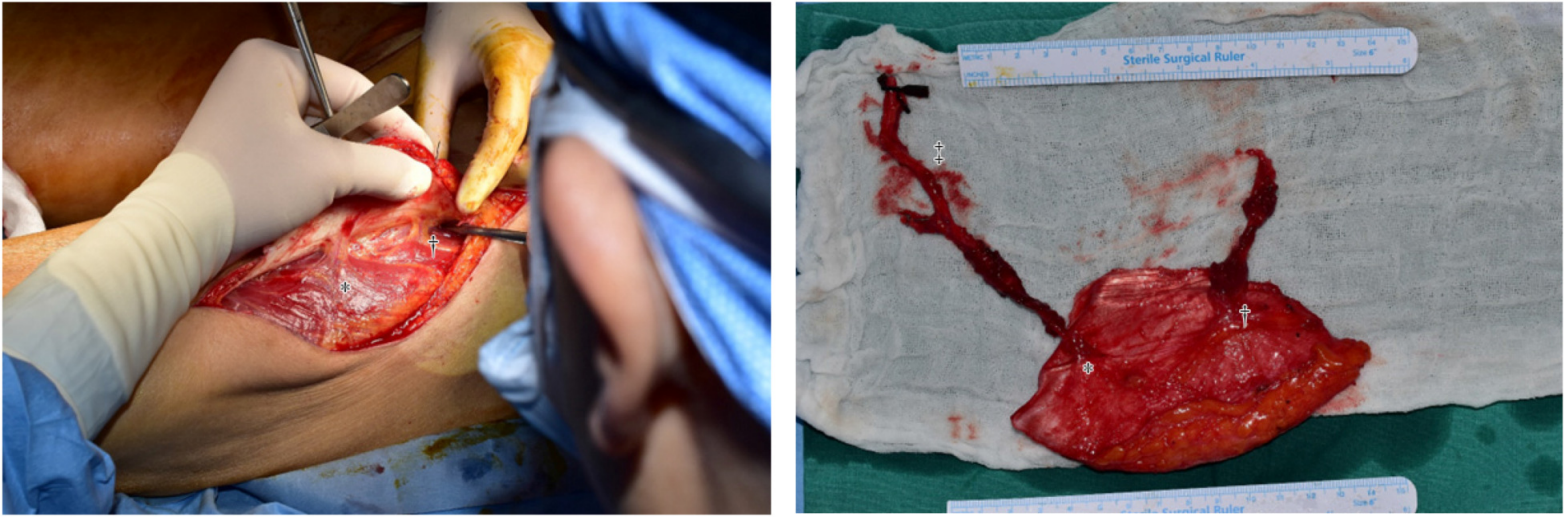
Intra operative photo. Two perforators and DB-LCFA are observed. * distal perforator; † proximal perforator; ‡ DB-LCFA. DB-LCFA descending branch of lateral circumflex femoral artery.

**Figure 3:**
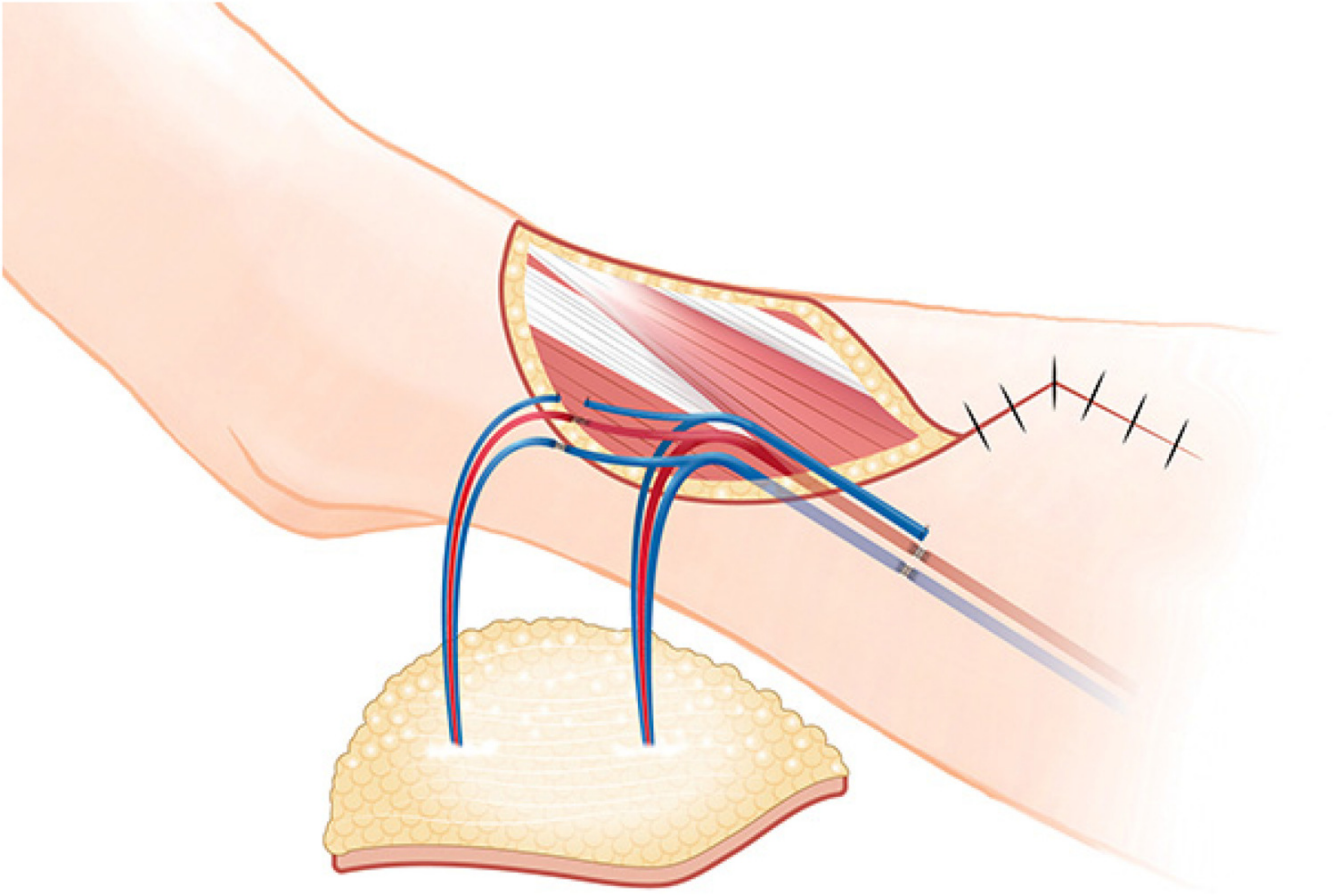
Schematic diagram of surgery. After pedicle anastomosis, the proximal perforator was anastomized with the distal end of DB-LCFA. DB-LCFA descending branch of lateral circumflex femoral artery.

After 5 days, ALT free flap was performed. It was decided to include two perforators of Flap and dissection was performed (Figure 2). The distal perforator was more reliable and descending branch of lateral circumflex femoral artery (DB-LCFA) was dissected, but the preserving proximal perforator was not connected to the DB-LCFA (Figure 2). The ATA of the recipient site and the proximal of DB-LCFA were anastomosed, but blood supply to the flap was not smooth. Salvage methods were tried to improve blood flow, but circulation in the flap was not observed. At this time, the author connected the remaining proximal perforator to the distal end of the DB-LCFA to secure additional blood supply. The blood supply improved, and the surgical site recovered without any complications (Figure 3).

## Discussion

Vascular compromise occurs because of hematoma, thrombosis, vessel spasm, vascular kinking, and technical errors (2, 3). If blood flow is not confirmed immediately after vessel anastomosis, arterial anastomosis is reopened, followed by heparin flushing, warm saline irrigation, and reanastomosis (4). However, if there is no improvement and it is determined that the perforator was damaged during the flap harvesting process, it may be necessary to abandon the old flap and re-harvest a new flap. In this case, various salvage methods were used, but circulation did not improve, and an attempt was made to directly connect the sparing perforator to recipient vessel to improve blood flow, but it could not be performed due to the difference in vessel diameter between sparing perforator of flap and ATA. Surgeon tried to find the side branch of DB-LCFA and connect it with the sparing perforator (5), but could not find a suitable side branch. At this time, the author modified the turbocharging method and connected the remaining perforator to the distal end of the DB-LCFA.

**Figure 4:**
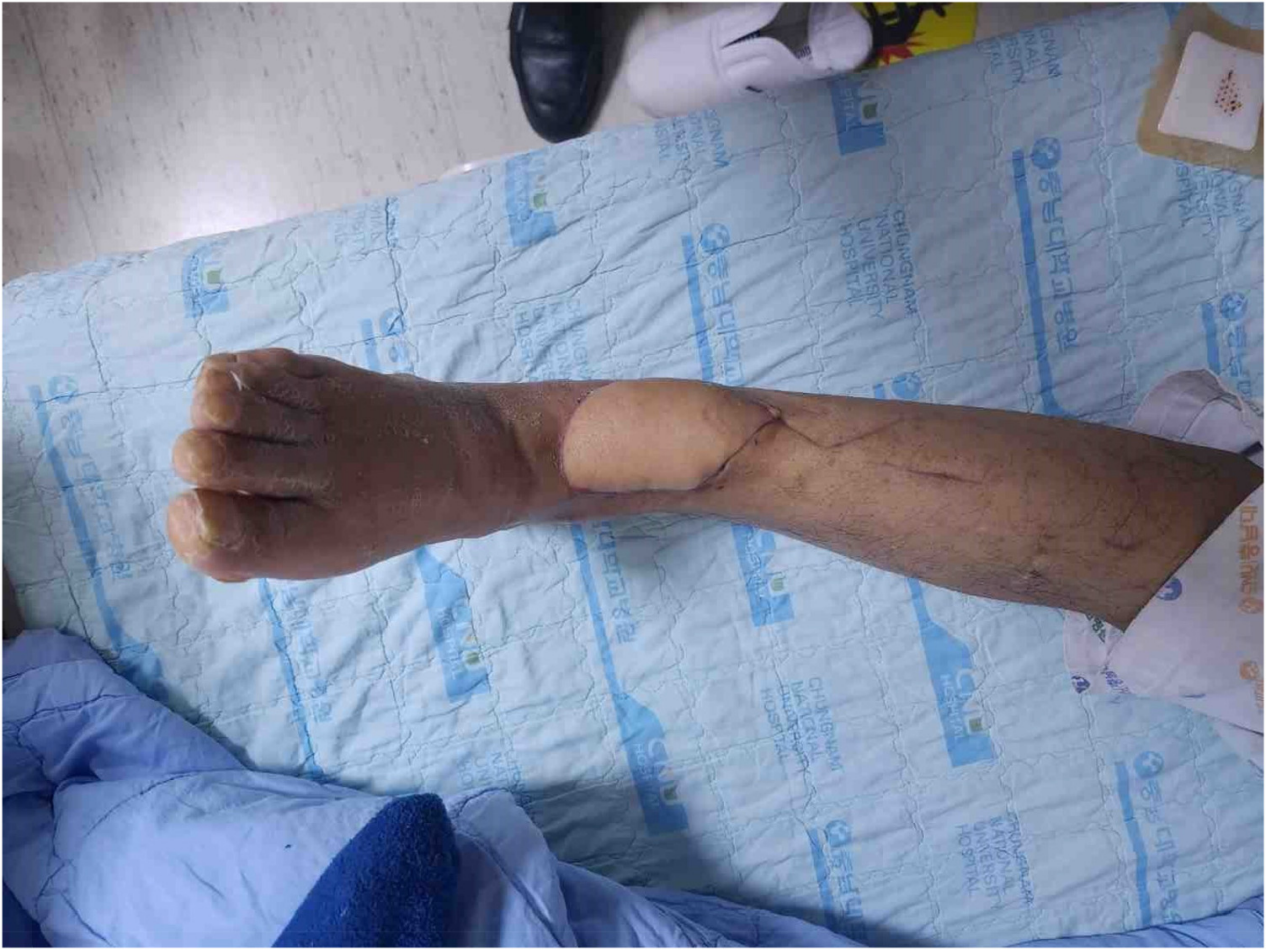
6 months later.

Supercharging and turbocharging are surgical techniques for enhancing blood flow to the flaps. Supercharging connects one of the supply vessels of a flap to the vessels outside the flap. In contrast, turbocharging involves interconnection of supplying vessels of a flap. In the ALT free flap, the turbocharging method connects another perforator to the side branch of the DB-LCFA (5). However, in this case, since there was no suitable side branch to connect the perforator, it was decided to modify the traditional turbocharging method and connect the distal end of the DB-LCFA to another perforator (Figure 4). After modified turbocharging, the blood supply of the flap improved, and the patient was able to recover safely.

In this patient, blood supply was not smooth with a single perforator and the main pedicle did not have an adequate side branch, which was thought to be because the patient had long suffered from diabetic mellites, hypertension, and diffuse atherosclerosis.

Preoperative peripheral angiography revealed that the artery (ATA) at the trauma site was damaged. However, compared to the trauma area, the proximal vessel was not blocked even though it had stenosis, so it was thought that it could be sufficiently used as a recipient vessel for the free flap. It has been reported that 5%–25% of patients undergo early re-occlosuion after peripheral angiography (6). In the case of this patient, a cardiologist was also consulted to perform a free flap before early re-occlusion, and free flap surgery was performed 5 days after the procedure.

A perforator is commonly observed in the proximal position of the perforator commonly used in ATL free flap surgery, but if it is not necessary for surgery, it is sacrificed during dissection. In the case of this patient, a proximal perforator was sparing, expecting that the arterial circulation of the flap might not be good due to severe atherosclerosis. I was more concerned about stenosis and spasm of the harvest vessel (LCFA-DB) and skin perforator, so I prepared a second perforator. During flap dissection, the second perforator was not connected to the descending branch of LCFA. In retrospect, it is assumed that it was connected to another branch (possibly the transverse branch) of LCFA (7).

After the reconstruction surgery, the patient underwent plate and screw removal surgery at an orthopedic surgery center 6 months later and is undergoing rehabilitation treatment. Recently, a patient came back for the first time in almost two years, and there were no problems with the surgical site. The patient has been followed up for two years to date. There have been no signs of late necrosis.

## Conclusion

If circulation is expected to be insufficient due to the large flap or atherosclerosis, using a modified turbocharging method by sparing the perforator is thought to be helpful for the success of the operation in ALT free flap.

## Data Availability

The original contributions presented in the study are included in the article/Supplementary Material, further inquiries can be directed to the corresponding author.
