# Correction: Anterolateral thigh free flap using modified turbocharging method: a case report

**DOI:** 10.3389/fsurg.2026.1804333

**Published:** 2026-03-13

**Authors:** Yooseok Ha, Donghyun Kim, Hyeokjae Kwon, Sunje Kim, Seung Han Song, Sang-Ha Oh, Joo-hak Kim, Ho Jik Yang, Hyunwoo Kyung

**Affiliations:** 1 Department of Plastic and Reconstructive Surgery, Chungnam National University Hospital, Daejeon, Republic of Korea; 2 Department of Plastic and Reconstructive Surgery, College of Medicine, Chungnam National University, Daejoen, Republic of Korea; 3 Department of Plastic and Reconstructive Surgery, Chungnam National University Sejong Hospital, Sejong, Republic of Korea

**Keywords:** turbocharging, anterolateral thigh, soft tissue defect reconstruction, perforator flap, salvage technique

An incorrect Funding statement was provided. An additional funder “Chungnam National University Hospital” was erroneously included. The correct funding statement reads:

“The author(s) declare financial support was received for the research, authorship, and/or publication of this article. This work was supported by the research fund of Chungnam National University.”

The original version of this article has been updated.

